# Gut and urinary microbiota: the causes and potential treatment measures of renal cell carcinoma

**DOI:** 10.3389/fimmu.2023.1188520

**Published:** 2023-06-27

**Authors:** Jian-wei Yang, Shun Wan, Kun-peng Li, Si-Yu Chen, Li Yang

**Affiliations:** Department of Urology, The Second Hospital of Lanzhou University, Lanzhou, China

**Keywords:** renal cell carcinoma, microbiome, immunotherapy, targeted therapy, gut microbiota, urinary microbiota, fecal microbiota transplantation, biomarker

## Abstract

Mounting evidence suggests that the gut microbiota plays a crucial role in the development and treatment of various cancers. Recent research on the urinary microbiota challenges the long-standing belief that urine is sterile, as urinary microbiota has been implicated in the development of bladder and prostate cancers, similar to the role of gut microbiota in cancer development. Although the precise involvement of microbiota in the proliferation and differentiation of renal cell carcinoma (RCC) remains unclear, dysbiosis is considered one possible mechanism by which microbiota may contribute to RCC development and treatment. This review summarizes potential mechanisms by which gut microbiota may contribute to the development of RCC, and provides evidence for the involvement of urinary microbiota in RCC. We also explore the role of gut microbiota in RCC treatment and propose that the composition of gut microbiota could serve as a predictive marker for the potential efficacy of immune checkpoint inhibitors (ICIs) in RCC patients. Additionally, evidence suggests that modulating the abundance and distribution of microbiota can enhance the therapeutic effects of drugs, suggesting that microbiota may serve as a promising adjuvant therapy for RCC. Overall, we believe that further investigation into the gut and urinary microbiome of RCC patients could yield valuable insights and strategies for the prevention and personalized treatment of RCC.

## 1 Introduction

Renal cell carcinoma (RCC) is a solid tumor that originates in the renal parenchyma and is resistant to chemotherapy. According to cancer statistics from 2017, RCC accounted for 4.1% of all newly diagnosed cancers, and its incidence has been on the rise (1). Currently, RCC is the sixth most common cancer in men and the tenth most common cancer in women. Moreover, up to 17% of patients are found to have distant metastasis at the time of diagnosis (2, 3).

Microbiota is a normal component of the human body’s ecology, with approximately 4×10^13^ bacterial microorganisms and up to 3×10^3^ species. Nearly 97% of these microorganisms reside in the colon and participate in normal human physiological processes, but they may also contribute to some pathological reactions (4). At the same time, numerous studies have demonstrated that dysbiosis of the microbiota may be a pathogenic factor for some tumors. For example, Helicobacter pylori-induced type B gastritis is associated with an increased risk of cancer, and Propionibacterium acnes has been found to induce inflammation and may be closely associated with the development of gastric and prostate cancers (5–8).

It is well known that the function of T cells in cancer patients and physiological immune responses against tumor-associated antigens (TAAs) are often suppressed by the interaction between immune checkpoints and their ligands. Immune checkpoint inhibitors (ICIs) regulate the dysfunctional immune system by blocking the interaction between cytotoxic T lymphocyte-associated protein 4 (CTLA-4) or targeting programmed cell death 1 (PD-1) and its ligand programmed cell death-ligand 1 (PD-L1), thereby inducing CD8-positive T cell killing of cancer cells (9). ICIs have played a critical role in treating kidney cancer, advanced melanoma, and non-small cell lung cancer patients. Recent studies suggest that the diversity of gut microbiota may be related to the efficacy of ICI therapy.

However, there is currently a lack of research on the role of microbiota in the occurrence and treatment of kidney cancer. Therefore, this review aims to provide new insights into the prevention and personalized treatment of this tumor by summarizing the relationship between gut and urogenital microbiota and the occurrence and treatment of renal cell carcinoma based on existing literature.

## 2 Description of microbial detection technologies

Next-generation sequencing (NGS) is widely acknowledged as a crucial method for detecting microbial communities (10). The main microbial detection technologies in NGS are shotgun whole-genome sequencing (WGS) and 16S rRNA amplicon sequencing. These techniques can be used to obtain taxonomic features of microbial populations by analyzing the complete DNA information of microorganisms collected using microbial DNA isolation kits. WGS separates all bacterial DNA sequences in a microbial community, aligns these sequences with a metagenomic database, and classifies the microbial population. 16S rRNA amplicon sequencing amplifies specific regions in the 16S rRNA gene through polymerase chain reaction (PCR) and identifies different bacterial communities by comparing relatively non-conserved sequences in the rRNA sequence database. Both WGS and 16S rRNA amplicon sequencing technologies provide two key parameters: alpha diversity and beta diversity. Alpha diversity describes the microbial community distribution within a single sample, while beta diversity describes the differences in microbial diversity between two different samples. While both techniques have their own advantages, 16S rRNA amplicon sequencing is more cost-effective than WGS. However, WGS provides higher accuracy and is better at describing microbial species richness (11). To mitigate potential degradation of microbial DNA caused by environmental factors, it is essential to promptly process biological samples. Additionally, both techniques should prevent reduced microbial DNA abundance resulting from sample storage, as this can negatively impact detection accuracy. In addition, in clinical samples such as body fluids and swabs, the high background of host DNA can also interfere with the detection of microorganisms (12). Using microbial enrichment and host gene depletion techniques to pretreat samples can also increase the sensitivity of WGS and compensate for its potential limitations (13, 14).

## 3 The relationship between renal cell carcinoma and microbiota

**Figure 1:**
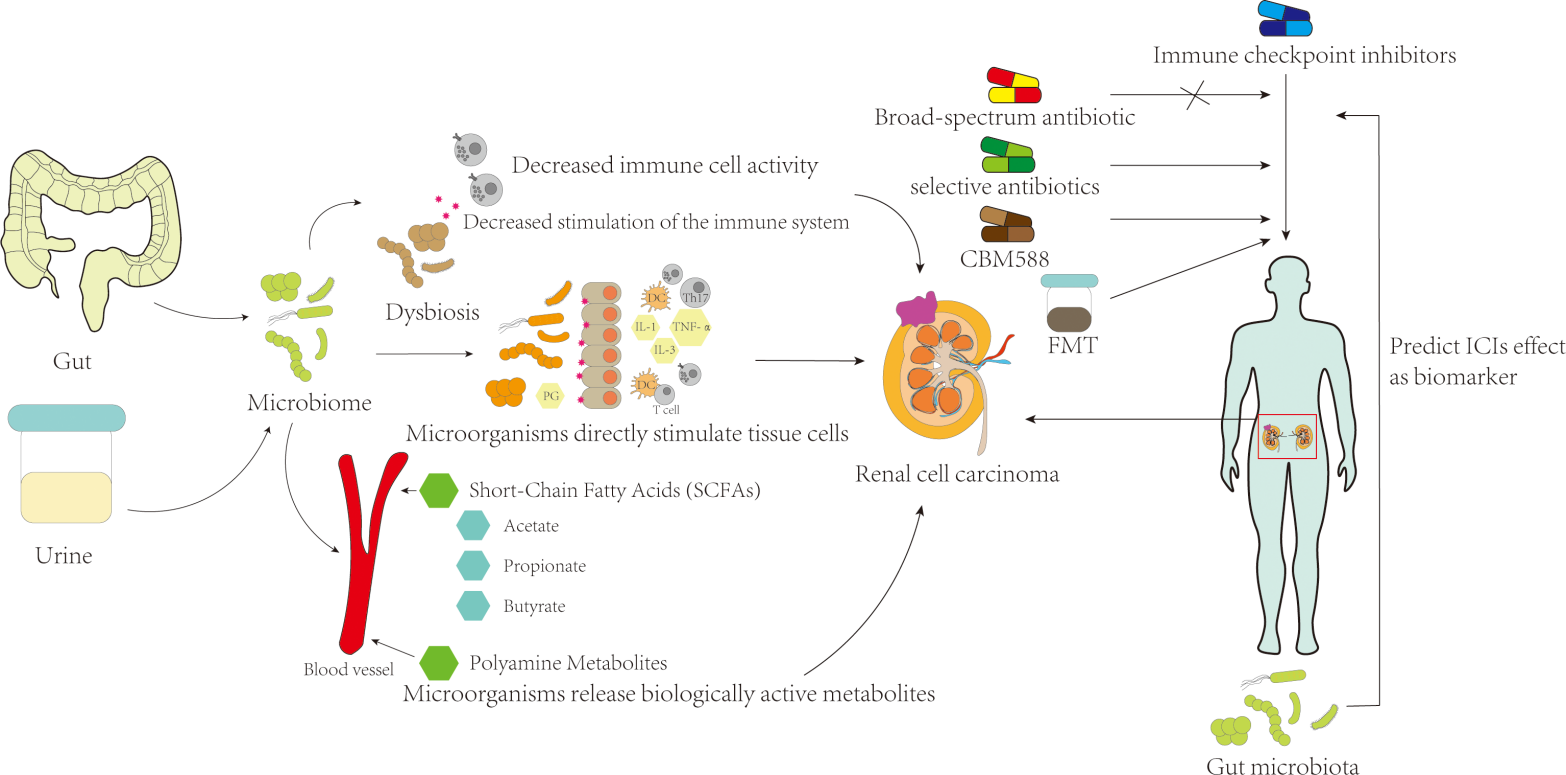
The mechanism of microbiome on renal cell carcinoma and its possible effect on treatment.

It is widely believed that the occurrence and development of RCC are the result of multiple factors, and the microbiota may act as a risk factor to promote RCC.Previous studies suggest that microorganisms can induce tumorigenesis through several primary mechanisms (1): pathogenic microorganisms directly interact with host tissue cells, causing tissue cell death and repair, and driving normal tissue cells to transform into tumor cells by affecting genome stability, anti-cell death, and proliferation signaling (15, 16) (2); pathogenic microorganisms can cause local tissue inflammation, and inflammatory molecules produced by inflammatory cells such as reactive oxygen species, reactive nitrogen species, cytokines, and chemokines can promote tumor growth and metastasis (17) (3); disruption of the microbial homeostasis can result in some bacteria or their components failing to trigger an effective host inflammatory response, while at the same time, some microorganisms can cause impaired activation of immune cells and subsequent immune defects to avoid destruction by the host immune system, thereby protecting tumors from immune cell attack (18–20) (4); bioactive metabolic products or secretions released by pathogenic microorganisms can cause changes in the living environment of host tissue cells, leading to the destruction of normal biological barriers of host cells, and these products can also regulate tumor occurrence through the host circulatory system away from the site of microbial growth (21–23). (**Figure 1**)

In a study conducted in China, Chen and colleagues investigated the gut microbiota composition of 51 ccRCC patients and 16 healthy individuals using 16S rRNA sequencing analysis. The study firstly revealed significant differences in the distribution of gut microbiota between the ccRCC patients and the healthy control group. The researchers also identified five bacterial taxa, including Blautia, Streptococcus, [Ruminococcus] torques group, Romboutsia, and [Eubacterium] hallii group, which were found to be widely present in the gut of ccRCC patients. These five bacterial taxa were able to accurately differentiate between ccRCC patients and healthy individuals with an AUC of 93.3%, suggesting that they could be potential biomarkers for ccRCC. Moreover, *in vitro* experiments of the study demonstrated that S. lutetiensis, a member of these microbial taxa, promoted the proliferation, migration, and invasion of ccRCC (24). In another study, Heidler et al. firstly used 16S rRNA sequencing to detect bacteria in the kidney tissues of 10 RCC patients who underwent laparoscopic nephrectomy and had no history of urinary tract infection in the past 6 months (25). Their study, along with those conducted by Liss and Wang et al., demonstrated that microbial communities not only existed in the kidney tissues but also showed significant differences in β-diversity between benign and malignant tissues (26, 27).

As the initial part of the digestive tract, the oral microbiota is closely associated with the gut microbiota. Epidemiological evidence and the detection of same microbiota in the oral and urogenital systems have recently led to the discovery of the oral-urogenital axis. It is believed that oral microbiota may contribute to the onset and progression of urogenital system malignancies. The ecological imbalance of the oral microbiome has been identified as the primary factor leading to the development of periodontitis (28). In addition, several studies have suggested a significant association between periodontitis and an increased risk of urological cancers such as prostate cancer (SIR: 3.75, 95%CI: 0.95-10.21) (29) and bladder cancer (HR=5.06, 95%CI: 2.32-11.0) (30). However, the relationship between periodontitis and kidney cancer remains controversial. Michaud et al. conducted a prospective cohort study of 48,375 men and followed up with them after eight years, revealing that periodontitis may increase the risk of developing kidney cancer (HR=1.49, 95%CI: 1.12-1.97) in the initial study and showed a similar trend in the follow-up analysis (HR=1.06, 95%CI: 0.61-1.85) (30, 31). However, it is important to note that studies by Nwizu, Wen, and Ma have reported no correlation between periodontitis and the incidence of kidney cancer (32–34). These inconsistent conclusions may be due to differences in the race and gender of the study populations, which could lead to variations in the oral microbiota. This underscores the importance of expanding sample sizes and data in the sample bank to further investigate the relationship between periodontitis and kidney cancer, given the diverse distribution of microbes.

The conventional belief that urine is a sterile body fluid has been challenged by recent research. It has been suggested that a reduction in the abundance and diversity of the urinary microbiome may increase the risk of urological tumors, including bladder and prostate cancer. Although the existing evidence does not allow us to infer causality, two hypotheses have been proposed to explain the relationship between the urinary microbiome and urological tumors. The first hypothesis is that the urinary microbiome may directly impact the development and progression of urological tumors, while the second hypothesis suggests that urological tumors may affect the diversity of the gut microbiome. Regardless of the scenario, significant alterations in the urinary microbiome of urinary system tumors compared to the normal population have been observed, suggesting the enormous potential of urinary microbiome as a non-invasive biomarker in the diagnosis, treatment, and prognosis of urinary system tumors. In a clinical study involving 12 patients with RCC, significant differences were observed in the urinary microbiota composition compared to healthy individuals (35). However, due to the current scarcity of clinical evidence directly demonstrating the relationship between the urinary microbiome and RCC, it is imperative that we further collect urine samples from both RCC patients and healthy control groups for clinical experiments. This will enable us to ascertain the differences in microbiome distribution between the two groups and confirm the relationship between the microbiome and RCC (36). Although direct clinical evidence demonstrating a causal relationship between urinary microbiome and RCC is scarce, it has been discovered that many urologic disorders resulting from urinary microbiome dysbiosis, such as urinary tract infections and kidney stones, can increase the risk of developing RCC. In a questionnaire survey of 372 RCC patients and 2,248 individuals in the general population, Parker et al. found that individuals with a history of kidney or bladder infection were more likely to develop RCC (OR=1.9, 95%CI:1.5, 2.5). Moreover, male smokers with a history of urinary tract infections had a significantly higher risk of RCC (OR=9.7, 95%CI:5.0, 18.1) (37). This view was further supported by Dhote, who summarized all possible risk factors for RCC (OR=1.2-1.7) (38). In a recent study, Gupta et al. put forward the concept that dysbiosis could contribute to the formation of kidney stones and subsequently increase the risk of developing RCC. This hypothesis sheds light on the association between urinary tract infections and RCC and suggests that the microbiome may play an active role in the development of RCC (39). Through the application of 16S rRNA sequencing on the gut microbiota of healthy individuals and kidney stone patients, researchers found a significant reduction in the gut microbiota diversity of the latter group. Furthermore, the abundance of pro-inflammatory bacteria was found to be substantially higher in kidney stone patients than in healthy individuals (40). The presence of urinary dysbiosis has also been observed to lead to significant structural differences in urinary microbiota between kidney stone patients and healthy individuals. Such differences in microbial composition may cause a potential increase in pro-inflammatory bacteria, resulting in the formation of kidney stones (41). Pol et al. established a correlation between the formation of kidney stones and an elevated risk of RCC (HR: 1.39, 95%CI 1.05-1.84), proposing that the chronic stimulation and recurrent infections caused by the stone may recruit inflammatory cells and promote cell damage and proliferation, thereby promoting the development of cancer (42).

## 4 Microorganisms and treatment of renal cell carcinoma

Statistics indicate that approximately one-third of patients with RCC who underwent targeted therapy eventually developed advanced disease. Prior to the advent of ICIs, tyrosine kinase inhibitors (TKIs), such as sunitinib, sorafenib, and bevacizumab, were used as first-line standard treatment, with a median survival time of 22 months (95% CI, 20.2-26.5 months) (43). Recent studies have shown that the use of ICIs, such as nivolumab plus ipilimumab, has resulted in better overall survival rates in advanced RCC patients as compared to TKIs. Additionally, patients treated with ICIs have reported more favorable treatment outcomes, such as fewer symptoms and improved Health-Related Quality of Life (HRQoL), compared to those treated with sunitinib (44, 45). However, despite the much higher overall survival time and response rate of ICIs or combination therapy compared to traditional TKIs, a considerable number of patients still develop primary resistance or do not respond, leading to tumor progression (46).

Recent studies have revealed that microbiota can modulate the therapeutic response of various tumors by regulating their own immune response. For instance, in colorectal cancer, the level of F. nucleatum has been found to increase and enhance the effectiveness of PD-L1 blockade (47). In addition, symbiotic Bifidobacterium has been shown to promote the efficacy of anti-PD-L1 in melanoma (48). As ICIs do not directly kill tumor cells and instead mediate their therapeutic effects by inducing T cell activation, it has been hypothesized that the microbiota, which plays a critical role in regulating systemic immune responses, may enhance the efficacy of ICIs in treating RCC. Current research on enhancing the efficacy of RCC drugs and predicting treatment response outcomes through microbiota has yielded promising results, indicating that the critical role of the microbiota in RCC is gradually being uncovered. However, the current focus of microbiota research in RCC treatment lies predominantly on the gut microbiota, while the study of the relationship between urinary microbiota and RCC treatment outcomes remains largely unexplored. This review will emphasize the relationship between the gut microbiota and RCC treatment, providing a theoretical foundation and research framework for future investigations on the correlation between urinary microbiota and RCC treatment.

### 4.1 The gut microbiome can affect kidney cancer treatment, and antibiotic use can impact the efficacy of ICIs

Multiple studies have shown that the microbiome can modulate the corresponding immune responses in the human body. Certain bacteria can enhance the therapeutic effects of drugs on tumors and exhibit anti-tumor capabilities by producing metabolites that induce immune responses or by directly counteracting the tumor microenvironment (TMEs) (49). Recent research on the microbial metabolite butyrate has shown that it can directly enhance the anti-tumor response of CD8 T cells by promoting the IL-8 signaling pathway and the ID2-dependent pathway (29). Furthermore, MAGER et al. found that the microbial metabolite adenosine enhances the therapeutic efficacy of ICIs in tumor treatment and may be utilized in the development of microbiota-based adjunctive therapies (50). Additionally, certain B vitamins produced by the microbiota, such as vitamin B6, are believed to effectively enhance cell-mediated immune responses, regulate T cell activation and differentiation (51, 52), and improve the therapeutic efficacy of chemotherapy drugs by inducing and strengthening anti-cancer immune responses (53). Research by Paulos et al. has also shown that microbial components or products, such as lipopolysaccharides, can promote dendritic cell activation and, in conjunction with TLR4 binding, enhance the anti-tumor function of CD8 T cells (54).

Numerous studies have highlighted the variation in gut microbiota distribution and abundance among RCC patients with different responses to treatment. While immune therapies targeting PD-L1 and PD-1 have revolutionized the treatment and prognosis of RCC, some patients remain unresponsive. Salgia et al. have found that patients who benefited from ICIs exhibited higher gut microbial diversity compared to those who did not (55). A comprehensive analysis of 44 cohorts showed that the use of antibiotics (ATBs) in ICIs-treated malignant tumors negatively correlated with ORR, PFS, and OS, and the duration of ATB usage might impact ICI efficacy (56). However, the use of ATBs did not have a similar effect on patients receiving mTOR inhibitors or VEGF-T therapy (57). Studies on non-small cell lung cancer, melanoma, digestive tumors, and RCC have shown that patients who did not receive ATB or proton pump inhibitors (PPIs) before ICIs had better PFS than those who did (58). Derosa et al. also observed that the usage of ATBs in advanced RCC patients receiving nivolumab monotherapy led to a significant reduction in the objective response rate. Moreover, their analysis of the gut microbiota revealed a correlation between dysbiosis and resistance to cancer immunotherapy in RCC patients (59). These comprehensive clinical studies have robustly established the significant influence of gut microbiota composition on the therapeutic effectiveness of ICIs in the context of RCC. Additionally, it has been firmly established that ATBs can impede the desired treatment outcomes in RCC patients. However, the potential impact of PPIs on the outcomes of ICI treatment in RCC patients remains inconclusive. Despite the findings by Giordan’s team, as mentioned earlier, suggesting that PPIs may diminish the therapeutic efficacy of ICIs, Mollica et al. conducted a study involving 71 patients and concluded that the use of PPIs does not impact the treatment outcomes of ICIs in RCC patients (58, 60). Notably, the negative impact of antibiotics on treatment efficacy is not universal. Hahn et al. found that prescribing antibiotics targeting Bacteroides spp. Improved the progression-free survival (PFS) of patients treated with VEGF-TKIs (61). During the study of VEGF-TKI targeted drugs, diarrhea was reported as a common side effect, with the highest incidence observed in patients using sunitinib (62). Recently, Su et al. found that the severity of sunitinib-induced diarrhea was negatively correlated with the diversity of the intestinal microbiota and the diversity of butyrate-producing bacteria, but positively correlated with the presence of Bacteroides spp (63).. These findings are consistent with the correlation between the use of antibiotics targeting Bacteroides spp. and improved PFS in RCC patients, as mentioned above. Based on the aforementioned evidence, it is highly plausible that the differential distribution of gut microbiota contributes to the varying therapeutic outcomes of ICIs. Additionally, ATB usage may disrupt the gut microbiota in ICI-treated RCC patients, leading to suboptimal treatment responses. This suggests that ATBs could serve as predictive factors for ICIs resistance. Furthermore, these findings inspire and caution us, indicating that modulation of the gut microbiota may offer a promising avenue to optimize the prognosis of RCC patients undergoing ICIs therapy. Hence, the prescription of ATBs should be approached with caution in advanced malignant tumor patients undergoing ICIs treatment.

### 4.2 Gut microbiota may serve as biomarkers for the efficacy and risk stratification of kidney cancer treatment

The human body is a complex organism that relies on various components, among which the gut microbiota plays a crucial role. The gut microbiome participates in the absorption of nutrients and also exerts immunoregulatory functions. Consequently, the gut microbiota is considered a potential pivotal component in immune surveillance against cancer and a guiding factor for immunotherapy of tumors (64). Iida et al. conducted an analysis of mice that were subjected to an antibiotic cocktail therapy and found that mice lacking gut microbiota suffered from severe immune deficiencies, ultimately leading to impaired efficacy of immune therapy which provides evidence for the correlation between gut microbiota composition and immunotherapy supported by animal experimentation (65). In studies of the use of ICIs to treat advanced melanoma, it has been found that the distribution of gut microbiota can serve as a biomarker to predict the response and ultimate outcome of ICI therapy for tumors (66). Similarly, in melanoma research, Frankel conducted a study using Metagenomic Shotgun Sequencing to investigate specific human gut microbial communities associated with the efficacy of ICIs. The study found that the previously mentioned Bacteroides spp. was enriched in the clinically beneficial group, suggesting that the microbiome may serve as a predictive biomarker for patient treatment outcomes (67). Concurrently, studies by VÉTIZOU and ROUTY also demonstrated the potential of the microbiome as a predictive biomarker for patient treatment outcomes (68, 69). In the treatment of renal cell carcinoma, patients who responded to ICIs had higher microbial diversity and distinct microbiota, as mentioned earlier. Therefore, it is worth exploring whether the characteristics of gut microbiota can be utilized to predict the therapeutic outcomes of kidney cancer patients in advance. This approach not only can prevent tumor progression and metastasis caused by ineffective treatment, but also benefit patients by changing treatment plans in a timely manner. **Table 1** summarizes the gut microbiota expression profiles in different clinical response groups to therapeutic agents, as observed in three studies investigating the relationship between ICIs and the efficacy of renal cancer treatment. Upon analyzing the high-expressing bacterial species in the clinical benefit group, it was observed that Akkermansia muciniphila (Akk) was highly expressed in the clinical benefit groups of ICIs in three study cohorts. Akk is an anaerobic bacterium that resides in the intestinal mucosal layer and uses intestinal mucin as the sole source of carbon and nitrogen required for survival (72). It also exhibits inhibitory effects on the growth of other mucin-degrading bacteria that are pathogenic (73). Akk has been shown to secrete substances that can inhibit tumor growth and increase treatment efficacy in multiple *in vitro* experiments (74, 75). Furthermore, the presence of Akk in the intestine can increase the microbial diversity of patients by promoting the growth of other symbiotic bacteria (76). Recent studies have suggested that Akk is associated with the clinical benefit of ICIs in non-small cell lung cancer (NSCLC) and can serve as a novel prognostic factor for NSCLC ICI treatment (77). However, the role of Akk in RCC is currently unknown, and further clinical trials are needed to confirm its relationship with the immunotherapy benefit in RCC. In addition, more samples should be collected to validate the potential of Akk as a prognostic and risk stratification biomarker for RCC.

**Table 1:** The distribution of gut microbiota between the clinical benefit group and clinical non-benefit group.

Reference	Research drug	Study subjects	Conclusion	Bacterial Groups with Clinically Beneficial Expression	Bacterial Groups without 1Clinically Beneficial Expression
Derosa (59)	Nivolumab	69 advanced renal cell carcinoma patients.	The composition of microbiota is influenced by TKIs and ATBs, which in turn affect the efficacy of ICIs.	A.muciniphila; Bacteroides salyersiae; Eubacterium siraeum	E.bacterium 2-2-44A; C.hathewayi; Clostridium clostridioforme
Salgia (55)	Nivolumab or Nivolumab plus Ipilimumab	31 patients with kidney cancer receiving medication.	There is a correlation between higher microbial diversity and better treatment outcomes	Bifidobacterium adolescentis; Barnesiella intestinihominis; Odoribacter splanchnicus; Bacteroides eggerthii; Akkermansia muciniphila(relative abundance increases)	Bacteroides ovatus; Eggerthella lenta; Fusicatenibacter saccharivorans; Flavonifractor_plautii
Routy (70)	PD-1/PD-L1 mAb	100 patients with non-small cell lung cancer (n=60) and renal cancer (n=40) who received PD-1 inhibitors.	ICIs resistance may be associated with dysbiosis of gut microbiota, and the use of ATB can impact treatment outcomes.	Akkermansia muciniphila; Firmicutes;Eubacterium sp.CAG:146; Lachnospiraceae; Erysipelotrichaceae bacterium 5-2-54FAA; Cloacibacillus porcorum	Parabacteroides distasonis; Bacteroides nordi; Blautia; Bacteroides clarus; Clostnidiales bacterium VE202-14; Firmicutes bactenum CAG 227
Dizman (71)	VEGF-TKI	21 patients with metastatic renal cell carcinoma.	Dietary interventions can influence the gut microbiota of patients with mRCC receiving VEGF-TKI therapy, and the composition of the gut microbiota can serve as a predictor of the clinical benefit level in mRCC patients undergoing VEGF-TKI treatment.	Akkermansia muciniphila; Faecalibacterium prausnitziil; Bacteroides caccae; Barnesiella intestinihominis; Eubacterium sp CAG 251; Roseburia faecis; Anaerostipes hadrus; Streptococcus salivarius; Streptococcus parasanguinis; Blautia coccoides ; Phascolarctobacterium faecium	Bacteroides vulgatus; Bifidobacterium longum; Lactobacillus vaginalis; Acidaminococcus intestine; Flavonifractor plautii; Actinomyces graevenitzii; Clostridium saccharolyticum; Bifidobacterium adolescentis; Bacteroides ovatus; Ruminococcus bicirculans; Eubacterium callanderi; Eubacterium eligens; Megasphaera sp MJR8396C; Acutalibacter muris

### 4.3 Gut microbiota as an adjuvant therapy for renal cell carcinoma

As our understanding of the microbiota and RCC continues to evolve, new insights and treatment strategies have emerged for preventing and managing RCC. Given the mounting evidence linking gut microbiome composition to ICI response, it is reasonable to explore methods for modulating the microbiota to promote a microbial composition that is more conducive to ICI response. This could involve maintaining gut microbial balance or selectively targeting individual microbes for modulation. As previously mentioned, the overuse of broad-spectrum antibiotics may reduce treatment efficacy in kidney cancer patients and exacerbate treatment side effects due to microbial imbalance. Nonetheless, by analyzing the gut microbiota of patients with varying treatment responses, we can identify specific microbial communities that are associated with favorable or unfavorable outcomes, and selectively deplete or augment these communities using targeted antibiotics or other interventions to improve treatment response and reduce side effects (78). Fecal microbiota transplantation (FMT) is a widely used therapeutic approach for managing several conditions, such as inflammatory bowel disease, metabolic disorders, Clostridioides difficile infection, and obesity. Since gut microbiota is considered a potential modulator of ICIs response, and the use of antibiotics has been found to weaken the efficacy of ICIs, it has been suggested that FMT could potentially transfer the beneficial gut microbiota from ICI responders to non-responders, supplementing beneficial bacteria and overcoming resistance. Clinical trials involving FMT have been conducted in melanoma patients, and it is exciting to note that among the enrolled cohort of 16 melanoma patients who exhibited resistance to anti–PD-1 therapy and received adjunctive FMT therapy, three individuals achieved objective responses (OR), while three others experienced sustained stable disease (SD) for over 12 months (79). This notable transformation can be attributed to the discernible modifications in the composition of gut microbiota in the resistant patients, subsequently exerting a profound influence on the intricate tumor microenvironment. In the context of RCC, studies have shown that FMT may possess similar capabilities to inhibit resistance to anti-tumor drugs, as observed in the aforementioned melanoma studies. In one experiment, fecal samples from nivolumab-responsive RCC patients were transplanted into RCC-bearing mice that were resistant to the drug. The results indicated that FMT effectively suppressed drug resistance in the mice and improved the function of mucosa-associated invariant T (MAIT) cells, which enhanced immunity against the tumor (59, 80). The findings of FMT suppressing drug resistance in RCC-bearing mice are promising, but its efficacy in treating RCC resistance in humans remains to be confirmed by further clinical trials. In a clinical trial involving 20 patients who experienced severe diarrhea after using tyrosine kinase inhibitors, the use of FMT was found to markedly alleviate diarrhea symptoms compared to a placebo group (81). Currently, FMT is only used to treat TKI-induced diarrhea caused by dysbiosis. Clinical studies are needed to establish the safety and efficacy of FMT for treating RCC resistance (82).

Newer evidence indicates that the consumption of probiotics may be linked to a reduced risk of adenomas, while a higher intake of yogurt may be associated with a decreased occurrence of colorectal tumors (83). Additionally, the combined use of Clostridium butyricum therapy (CBT) with ICIs has been found to have a positive impact on the therapeutic efficacy of lung cancer patients (84). Considering the potential benefits of manipulating the gut microbiota to enhance the response to ICIs in RCC, CBM588, a live bacterial supplement containing butyrate-producing clostridia, was investigated as an adjunct therapy. Previous research has shown that CBM588 can effectively increase the abundance of Bifidobacterium spp in the intestine (85), which may have a positive impact on the efficacy of ICIs in RCC treatment. The results of the study showed that the addition of CBM588 as a live bacterial supplement to the standard ICIs treatment for RCC led to a significant increase in the abundance of live Bifidobacterium spp in the gut microbiome. Moreover, patients who received CBM588 as an adjunct to ICIs had a significantly longer progression-free survival compared to those who received ICIs alone (12.7 months vs 2.5 months, HR: 0.15, 95% CI 0.05-0.47, P < 0.001). Although the sample size was limited and the difference was not statistically significant, there was a higher response rate in patients who received CBM588 (20% vs 0%, P = 0.6588) (86). Moon and colleagues discovered that yeast extracts can effectively suppress the growth of RCC cells *in vitro* by regulating iron metabolism. These findings suggest that yeast extracts may have potential as an adjunct therapy for RCC (87).

FMT and live bacterial supplementation have shown potential in treating various cancers by improving the response efficiency of ICIs and reducing treatment-related side effects. Despite these promising findings, the efficacy of FMT and live bacterial supplementation in RCC patients has yet to be supported by sufficient clinical evidence. Further clinical trials are needed to establish the effectiveness of these methods in treating RCC.

## 5 Unresolved issues

(1) Current studies are predominantly based on case-control designs, which may potentially introduce selection bias. Moreover, given the limited sample size in current RCC research and the potential confounding factors such as diet, social environment, age, ethnicity, and geographical location that may influence microbiome analysis, large-scale prospective studies with robust control of confounding variables would provide the most convincing evidence for establishing the relationship between gut and urinary microbiome and RCC. This direction is of utmost importance for future research in this field.
(2) The inconsistency of reference databases currently poses a challenge for microbial population identification through 16S rRNA and WGS sequencing. Complementing sequencing data with proteomics and metabolomics can enhance the accuracy of microbial detection, thereby improving future research outcomes.
(3) Differences in urinary microbiota composition have been observed in patients with bladder and prostate cancer, suggesting a potential role in cancer pathogenesis. Urinary tract infections have also been linked to the occurrence of kidney cancer. However, current evidence on the relationship between urinary microbiome and kidney cancer is limited to one study involving only 12 patients. Further large-scale studies are required to confirm this hypothesis.
(4) The gut microbiome has shown potential as an adjuvant therapy in RCC immunotherapy, but its mechanism of action remains incompletely understood due to the microbiome’s diversity and individual variations, as well as the complex microbial interactions.
(5) Akk has demonstrated the ability to inhibit tumor growth in various *in vitro* cancer models. Moreover, it is a dominant bacterial species found in the gut of ICI responders, and understanding its mechanisms of action may offer potential strategies for reversing ICI non-responsiveness in RCC.

## 6 Summary

With the advancement of technology, the types and numbers of microbiota are no longer a constraint on human exploration. Increasing evidence from studies on human gut microbiota suggests that gut microbiota is involved in regulating various pathological and physiological processes. The microbiome represents a new field in oncology, and our understanding of it is gradually deepening and expanding. The possibility of using microbiota for cancer treatment and prevention is also increasing. In this review, we summarize the relationship between the microbiome and RCC development, as well as the relationship between gut microbiota and treatment, providing a theoretical basis and ideas for further exploring the role of the microbiome as a pathogenic factor in RCC and as a potential modulator of ICIs response in RCC patients through prospective clinical and animal experiments. Based on the existing clinical evidence, we believe that the gut and urinary microbiota may play a promoting role in the development and progression of RCC. However, due to the interactive relationship between microorganisms and the initial stage of research on urinary microbiota in RCC patients, there is an urgent need for randomized controlled trials to explore the causal effects of these hypotheses. Overall, further exploration of the microbiome can increase our understanding of the mechanism of RCC development, which is crucial for providing personalized treatment options for patients, increasing patient sensitivity to treatment, and ultimately increasing clinical benefits.

## Author contributions

The manuscript was conceived by LY, J-WY, SW, and K-PL. J-WY wrote the majority of the manuscript, while SW, K-PL, and S-YC contributed to the writing. J-WY, SW, and K-PL designed and drew the figures and tables. All authors contributed to the article and approved the submitted version.
